# Cancer Stem Cells in Small Cell Lung Cancer Cell Line H446: Higher Dependency on Oxidative Phosphorylation and Mitochondrial Substrate-Level Phosphorylation than Non-Stem Cancer Cells

**DOI:** 10.1371/journal.pone.0154576

**Published:** 2016-05-11

**Authors:** Cuicui Gao, Yao Shen, Fang Jin, Yajing Miao, Xiaofei Qiu

**Affiliations:** 1 Department of Pathology, Tianjin Medical University, Tianjin, China; 2 Department of blood transfusion, Tianjin Medical University General Hospital, Tianjin, China; 3 Research Center for Basic Medical Science, Tianjin Medical University, Tianjin, China; Instituto Nacional de Cardiologia, MEXICO

## Abstract

Recently, targeting cancer stem cells (CSCs) metabolism is becoming a promising therapeutic approach to improve cancer treatment outcomes. However, knowledge of the metabolic state of CSCs in small cell lung cancer is still lacking. In this study, we found that CSCs had significantly lower oxygen consumption rate and extracellular acidification rate than non-stem cancer cells. Meanwhile, this subpopulation of cells consumed less glucose, produced less lactate and maintained lower ATP levels. We also revealed that CSCs could produce more ATP through mitochondrial substrate-level phosphorylation during respiratory inhibition compared with non-stem cancer cells. Furthermore, they were more sensitive to suppression of oxidative phosphorylation. Therefore, oligomycin (inhibitor of oxidative phosphorylation) could severely impair sphere-forming and tumor-initiating abilities of CSCs. Our work suggests that CSCs represent metabolically inactive tumor subpopulations which sustain in a state showing low metabolic activity. However, mitochondrial substrate-level phosphorylation of CSCs may be more active than that of non-stem cancer cells. Moreover, CSCs showed preferential use of oxidative phosphorylation over glycolysis to meet their energy demand. These results extend our understanding of CSCs metabolism, potentially providing novel treatment strategies targeting metabolic pathways in small cell lung cancer.

## Introduction

Small cell lung cancer (SCLC) is a type of highly aggressive tumor which represents about 15% of all lung cancer cases [1,2]. Although patients with SCLC have an initial good clinical response to chemo- radiation therapy, most patients treated with these approaches will relapse after a short period[3]. This can in part be attributed to failure to eradicate cancer stem cells (CSCs), which have the ability to self-renew, to differentiate into multiple lineages and to initiate tumors in immunocompromised mice[4,5]. CSCs are believed to be more resistant to radio- and chemo-therapy than the non-stem cancer cells[5]. Therefore, it is crucial to develop promising therapeutic strategies targeting CSCs by overcoming their drug resistance.

Recently, it appears increasingly clear that the “metabolic reprogramming” of cancer cells has been an emerging hallmark of the cancer phenotype [6,7]. Unlike normal cells, cancer cells adopt an alternative metabolic pathway and exhibit enhanced glucose metabolism and production of lactate even in the presence of oxygen [8–10]. This preferential use of aerobic glycolysis[11], is known as the Warburg effect. Although aerobic glycolysis is thought to be a near-universal phenomenon in cancer cells, metabolic features of CSCs and their relevance in cancer therapeutics remain still controversy[12]. Ciavardelli et al [13] have reported that breast cancer stem cells is more glycolytic than their non-stem counterparts. The study by Liao [14] and his colleagues also has shown that ovarian cancer stem-like cells predominantly metabolize glucose by anaerobic glycolysis and pentose cycle. Meanwhile, Yuan et al [5] have shown that glioblastoma stem cells (GSCs) exhibit preferential use of glycolysis over mitochondrial respiration. However, Vlashi et al [15] have indicated that GSCs rely more on oxidative phosphorylation (OXPHOS) than glycolysis. Lagadinou et al[16] also have demonstrated that CSCs showed a greater reliance on OXPHOS for energy supply in leukemia cells. Pastò et al[9] have shown that cancer stem cells from epithelial ovarian cancer patients exhibited a metabolic profile dominated by OXPHOS. Although limited published data exist regarding metabolic properties of CSCs[17], none in SCLC. Therefore, to design novel therapeutic approaches that target metabolic pathways of CSCs in SCLC, profound knowledge of the metabolic state of this cell subpopulation is urgently needed[7].

To explore the metabolic properties of CSCs, the first mission is enrichment for CSCs in SCLC cells. Isolation of CSCs both in vivo and in vitro relies on specific surface biomarkers which facilitate sorting of cancer cells into phenotypically distinct subpopulations [18]. Urokinase-type plasminogen activator receptor (uPAR) is a glycosylphosphatidylinositol (GPI)-anchored protein [19] and is usually upregulated in multiple types of cancers [20]. Importantly, our work and that of others has identified uPAR as a mediator of cancer stem cell function [21,22]. For instance, uPAR^+^ cells in SCLC cell lines showed multidrug resistance and enhanced clonogenic activity in vitro compared with uPAR^-^ cells [23]. Previous work from our laboratory also have showed that the stem-like cell subpopulations may be enriched in the uPAR^+^ cells [24]. Therefore, we used uPAR sorting to enrich for CSCs in SCLC cell line H446.

In this study, we first compared the metabolic state of CSCs with that of non-stem cancer cells and found that CSCs were metabolically inactive tumor subpopulations which resided in a state showing low metabolic activity. Then we studied the major energy-producing pathways of CSCs in SCLC. Unlike non-stem cancer cells, CSCs showed preferential use of OXPHOS over glycolysis to meet their energy demand. In addition, we also found that CSCs could produce ATP through mitochondrial substrate-level phosphorylation.

## Materials and Methods

### Cell culture

The SCLC cell line NCI-H446 was obtained from the American Type Culture Collection (ATCC). These cells were cultured in RPMI-1640 (Biological Industries) supplemented with 10% FBS (Thermo Scientific HyClone), 100U/ml penicillin and 100μg/ml streptomycin in humidified air at 37°C with 5% CO_2_.

### Flow cytometry analysis

Cell sorting was performed on single cell suspension from H446 cells stained with primary antibody against uPAR (Mouse monoclonal antibody, 1:100, American Diagnostica, No. 3936). Then, the cells were washed and incubated with FITC-conjugated secondary antibody (Dako) for 30min. All samples were measured using a FACSCalibur flow cytometer (BD Biosciences) and analyzed with CellQuest software (BD Biosciences). uPAR^+^ cells and uPAR^-^ cells sorted by FACS were used in all of our experiments (S1 Fig).

### Oxygen consumption and extracellular acidification rate

The Extracellular Flux Analyzer allows for analyzing oxygen consumption rate (OCR) and extracellular acidification rate (ECAR) of a defined number of cells in real time and for monitoring their response to drug treatment[15]. Cells were plated at a density of 1×10^5^ cells each well of 24-well plates. The following day the cells were washed and fresh media was added. The cartridge was loaded to dispense three metabolic inhibitors sequentially as specific time points: oligomycin (inhibitor of ATP synthase, 1μM), followed by FCCP (a protonophore and uncoupler of mitochondrial OXPHOS, 0.3μM), followed by the addition of a combination of rotenone (mitochondrial complex I inhibitor, 1μM) and antimycin (mitochondrial complex III inhibitor, 1μM). Basal OCR and ECAR were measured, as well as changes in ECAR caused by the metabolic inhibitors described above. Several parameters were deducted from changes in OCR, such as basal OCR, maximum mitochondrial capacity, and mitochondrial reserve capacity (= [maximum mitochondrial capacity] − [basal OCR]) as described previously [15,25].

### Glucose uptake and lactate production measurements and calculation of bioenergetic organization

For measurement of glucose uptake, sorted cells were plated at a density of 1×10^6^/mL/well in 6-well plates and incubated in glucose-free RPMI 1640 for 2h at 37°C. After the addition of 2-[N-(7-nitrobenz-2-oxa-1,3- diazol-4-yl)amino]-2-deoxy-D-glucose (2-NBDG) to a final concentration of 100μM, the cells were incubated for an additional 1h, washed twice with PBS, and analyzed by flow cytometry [26]. L-Lactate production was detected using an L-lactate assay kit (Eton Bioscience, San Diego, USA) as previously described [27] and according to manufacturer’s instructions and L-lactate levels were normalized to cell numbers. The bioenergetic organizations of the cells were calculated as described in the report of Hao et al, we used the following: Lac(c) = lactate concentration in the control medium after 6h incubation; Lac (o) = lactate concentration in the medium after 6h of incubation with 2 μg/ml oligomycin; Glycolysis% = Lac(c) ×100/ Lac (o); and OXPHOS% = 100 − Glycolysis% [28].

### ATP assays

Cellular ATP concentration was measured using the ATP-based CellTiter-Glo Luminescent Cell Viability kit (Promega, Madison, WI) modified from the manufacturer’s protocol. Briefly, the cells were plated in 96-well plates at different densities. Three hours later, an equal volume of a single one-step reagent provided by the kit was added to each well and rocked for 10min at room temperature. Cellular ATP content was measured using a luminescent plate reader. To test the ATP depleting effect of the metabolic inhibitors, various concentrations of compound were added to the cells seeded at 1×10^4^ cells/well for additional 3h, and cellular ATP content was measured as described above.

### MTT assay

Cells were seeded in 96-well plate at 3000 cells per well in 100μl cell culture medium and incubated at 37°C for 24h and then treated with indicated compounds at various concentrations. After 72h incubation, the cells were incubated with 10μl MTT (at a final concentration of 0.5 mg/ml) at 37°C for 4 h [29]. The medium was removed and the precipitated formazan was dissolved in 100μl DMSO. After shaking for 10min, the absorbance at 490nm was detected using TECAN GENios Pro (BioTek Instruments).

### Tumor sphere-forming assay

To obtain spheres in culture, uPAR^+^ cells were treated with indicated compounds for 3 h. Then 1×10^3^ cells were seeded in serum-free medium (SFM) (DMEM/F12) supplemented with 20 ng/ml of basic fibroblast growth factor (bFGF) and epidermal growth factor (EGF) (PeproTech), 5 μg/ml of insulin (Sigma—Aldrich), 0.4% bovine serum albumin (BSA, Invitrogen), and 0.02 × B27 (Invitrogen). Culture medium was replaced or supplemented with additional growth factors twice a week. The total number of tumor spheres was counted after 14 days of culture.

### Tumorigenicity assay in nude mice

Animal experiments were performed on four weeks old BALB/cA nude mice purchased from Beijing HFK Bio-Technology. Co, LTD (Beijing, China). The protocols were performed after approval from the Committee on the Ethics of Animal Experiments of Tianjin Medical University (Permit Number: TMHaMEC2014030). The sorted uPAR^+^ cells were treated with 2μg/ml oligomycin for 24 h, washed, and harvested for subcutaneous inoculation on the left flanks of BALB/cA nude mice. The same numbers of control cells were inoculated on the right flanks of the same mice subcutaneously. Each inoculation site was injected with 5×10^5^ cells. The mice were then monitored for tumor formation without further drug treatment in vivo. Mice were observed 2–3 times per week by laboratory personnel and monitored for signs of distress (i.e., changes in appearance, respiration, activity, etc.). Tumors were measured once a week. All the animals in both groups were still in good condition until the end of the experiment. None of the animals required euthanasia prior to the experimental endpoint. After six weeks, the experimental animals were anesthetized with sodium pentobarbital (45mg/kg, intraperitoneal injection) and killed by cervical dislocation. Tumor volume = 0.5 × length× width^2^.

### Electron microscopy

uPAR^+^ cells and uPAR^-^ cells were sorted by FACS as described above. The ultrastructural analysis of sorted cells was performed as described in the report of Varum et al [30].

### Statistical analysis

Data was analyzed using SPSS for Windows version 17.0 software (SPSS Inc, Chicago, IL, USA). Each experiment was performed in at least three independent trials. All results are expressed as means ± s.e.m. and statistical comparisons of the data sets were analyzed using the unpaired Student’s t-test or the ANOVA test. P<0.05 was considered statistically significant.

## Results

### Extracellular flux analysis revealed the metabolic differences of CSCs and non-stem cancer cells

**Fig 1:**
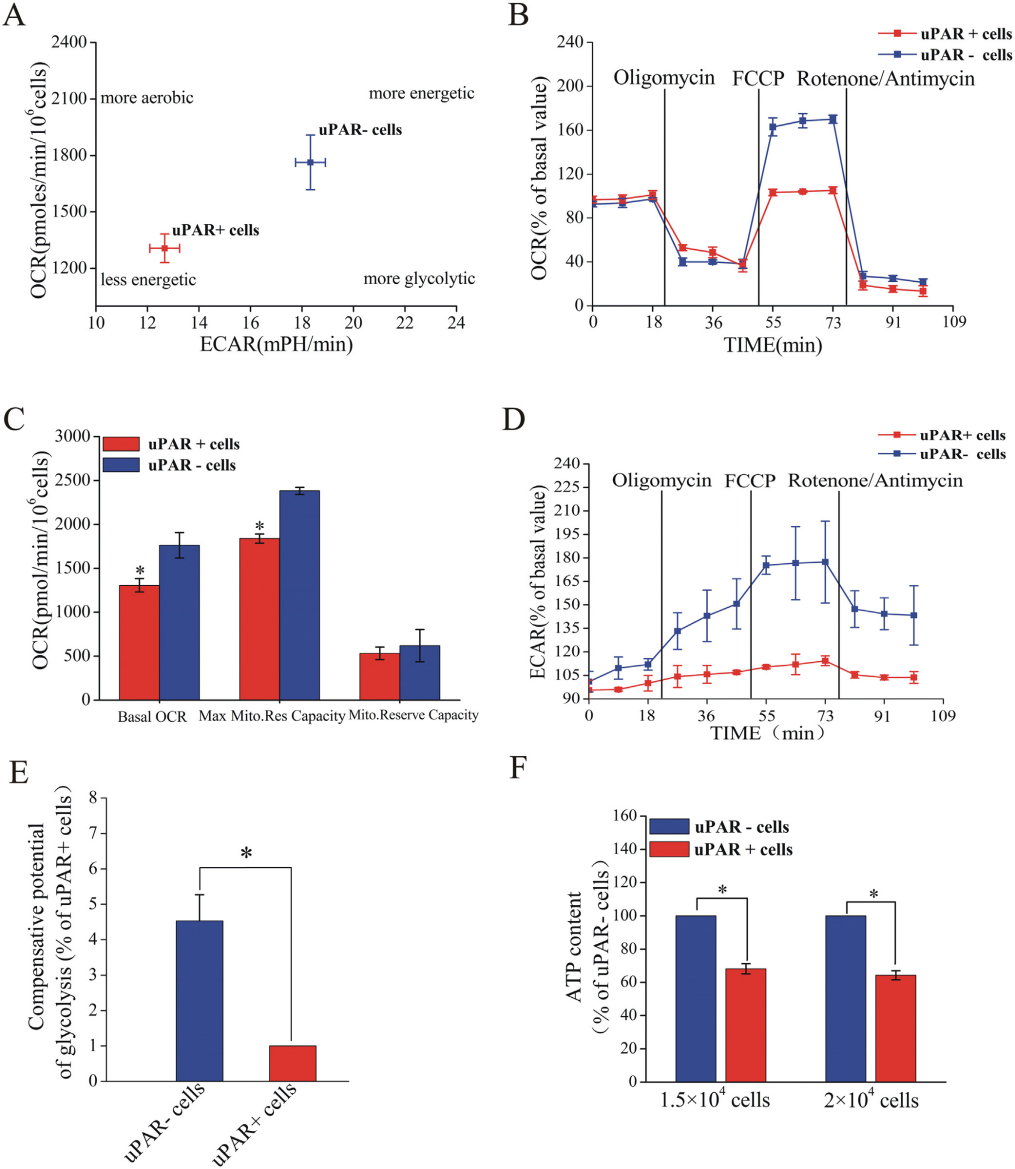
Bioenergetic analyses of CSCs and non-stem cancer cells in SCLC cell line H446. (A) Basal OCR and ECAR for uPAR^+^ and uPAR^-^ subsets. (B) The OCR after treatment with indicated inhibitors in uPAR^+^ cells versus uPAR^-^ cells. (C) Maximum mitochondrial respiratory capacities and mitochondrial reserve capacities in two subpopulations. (D) The ECAR after treatment with indicated inhibitors in uPAR^+^ cells versus uPAR^-^ cells. (E) The compensative potential of glycolysis of uPAR^+^ cells and uPAR^-^ cells. (F) ATP content in uPAR^+^ cells and uPAR^-^ cells. Data are expressed as mean ±s.e.m. of three independent experiments. Student’s t-test was used to calculate statistical significance. *P<0.05.

To gain insights to the metabolic profile of CSCs and to compare it with the non-stem cancer cells, we first performed extracellular metabolic flux analysis [16]. The OCR and ECAR were measured. As shown in Fig 1A, CSCs-enriched uPAR^+^ cells showed a lower basal OCR, indicative of lower mitochondrial respiration, as compared with their uPAR^-^ counterparts. When basal ECAR of uPAR^+^ cells and uPAR^-^ cells were compared, the uPAR^+^ cells was also less active in regard to fermentative glycolysis. To study the metabolic properties of two subpopulations in detail, we used a pharmacological profiling strategy, by combining the application of three metabolic inhibitors (oligomycin, FCCP, a combination of rotenone and antimycin) [30]. Treatment by oligomycin resulted in decrease in OCR of two cell subpopulations. Nonetheless, uPAR^+^ cells exhibited a less pronounced decrease in OCR compared with uPAR^-^ cells (Fig 1B). Uncoupling of OXPHOS using FCCP increased OCR to maximum in both cell subpopulations, with a less pronounced response being observed in uPAR^+^ cells (Fig 1B and 1C). The difference between FCCP OCR and basal OCR is a good indicator of respiratory ability (also referred to as the mitochondrial reserve capacity) of these cells[30]. Though the basal OCR of uPAR^+^ cells was lower, the mitochondrial reserve capacity of uPAR^+^ cells was comparable to that of uPAR^-^ cells (Fig 1C). Even if the basal ECAR is low in uPAR^+^ cells, it is possible that uPAR^+^ cells might upregulate glycolysis when mitochondrial OXPHOS is blocked [16]. To study the possibility, we measured the ECAR of these cells treated with OXPHOS inhibitors, which is a parameter of compensative potential of glycolysis[16]. Intriguingly, uPAR^+^ cells showed a significantly decreased compensative potential of glycolysis when mitochondrial energy production was inhibited (Fig 1D and 1E). Moreover, our results showed that uPAR^+^ cells had decreased ATP levels compared to uPAR^-^cells (Fig 1F). Taken together, our data suggest that CSCs-enriched uPAR^+^ cells are metabolically inactive cell populations in SCLC.

### CSCs showed less glucose uptake and lactate production in SCLC

**Fig 2:**
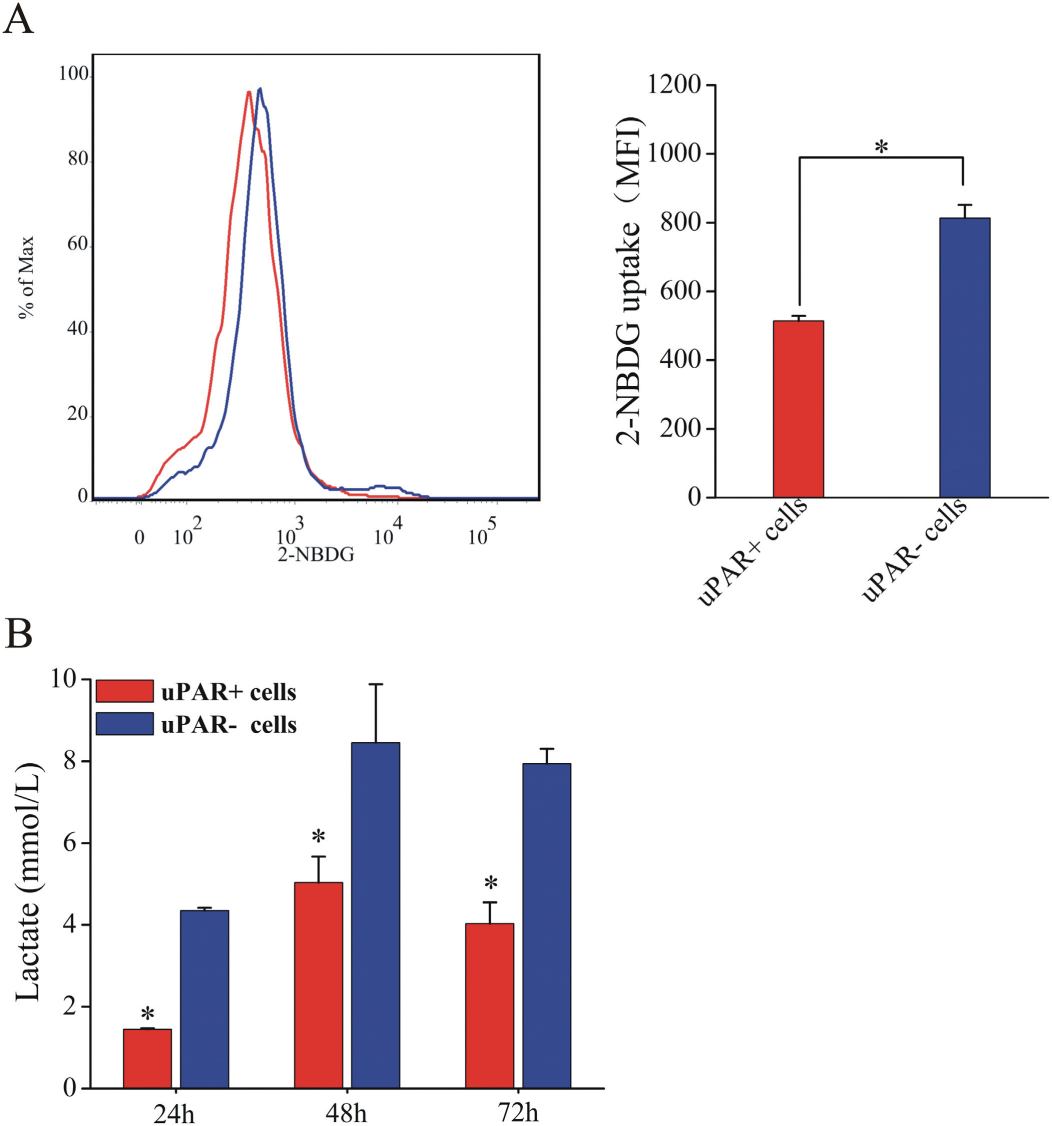
Glucose uptake and lactate production in CSCs and non-stem cancer cells. (A) Glucose uptake by uPAR^+^ cells and uPAR^-^ cells using 2-NBDG. Left, histogram representation of 2-NBDG intensity. Right, quantification of 2-NBDG uptake as difference in mean fluorescence intensity (MFI) between samples and controls. (B) Lactate production rates of uPAR^+^ cells and uPAR^-^ cells. Data are expressed as mean ±s.e.m. of three independent experiments. Student’s t-test was used to calculate statistical significance. *P<0.05.

To further investigate the metabolic differences of uPAR^+^ cells and uPAR^-^ cells, we evaluated the glucose uptake of two subpopulations. uPAR^+^ cells displayed less glucose uptake than uPAR^-^ cells (Fig 2A). Our ECAR measurements have showed differences in the lactate generation between uPAR^+^ cells and uPAR^-^ cells (Fig 1A and 1D). Consistent with these findings, uPAR^+^ cells exhibited significant decreased levels of lactate production compared with the uPAR^-^ cells (Fig 2B). This again indicated that CSCs were less glycolytic than their non-stem cancer cells. Glutaminolysis is usually active in tumor cells, therefore we measured the 2-deoxyglucose-sensitive lactate production in uPAR^+^ cells and uPAR^-^ cells. As shown in S2 Fig, 2-DG caused a substantial reduction of lactate production. These results suggested glutaminolysis was not so active in small cell lung cancer cell line H446 as in other tumor cells.

### Mitochondrial number and morphology of CSCs was different from that of non-stem cancer cells

**Fig 3:**
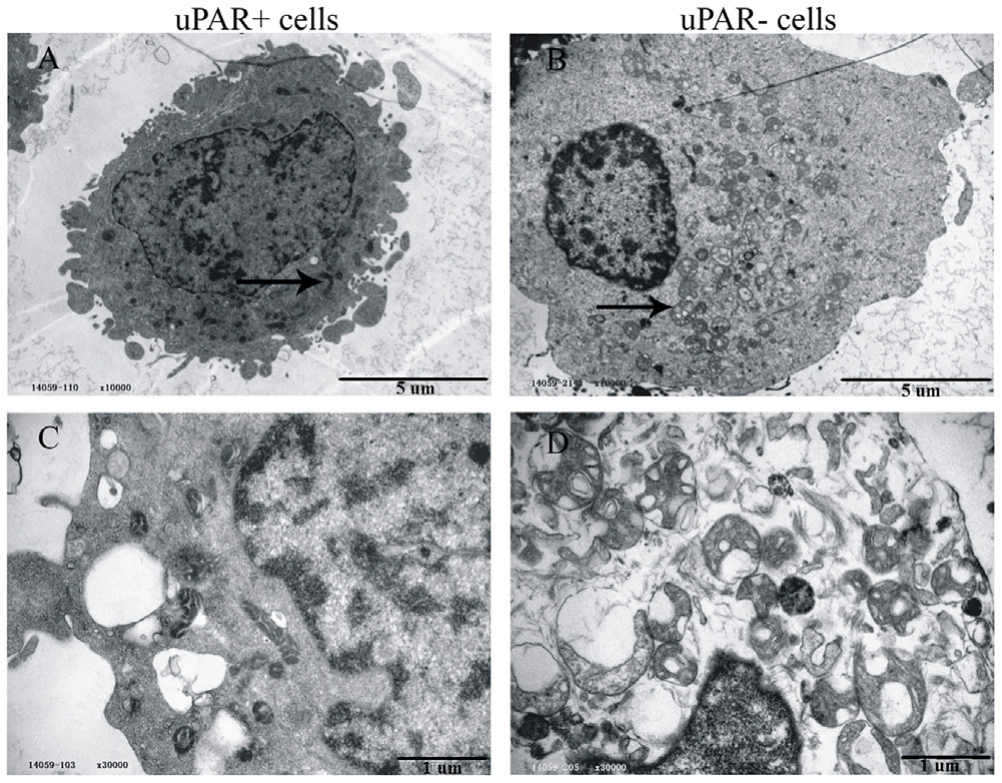
Numbers and morphology of mitochondria in CSCs and non-stem cancer cells. Ultrastructural analysis of sorted uPAR^+^ cells and uPAR^-^ cells using transmission electron microscopy. Representative images are shown at magnification of ×10,000 in upper panels and ×30,000 in lower panels. Arrows in lower panel indicate examples of mitochondria. Scale bars: 5 μm (upper panels) and 1 μm (lower panels).

Decreased mitochondrial respiration in uPAR^+^ cells could be attributed to lower numbers of mitochondria or immaturity of mitochondria in these cells compared with uPAR^-^ cells. To investigate this issue, we performed transmission electron microscopy [30] for uPAR^+^ cells and uPAR^-^ cells. As shown in Fig 3, the majority of mitochondria in uPAR^-^ cells were rounded to oval, displaying sparse and irregular cristae and an electron-lucent matrix. However, uPAR^+^ cells preferentially possessed activated mitochondria, including clustering of mitochondria, thickened cristae, and “wagon wheel” morphology [17,31]. This morphological assessment suggests that the mitochondria of uPAR^+^ cells are more mature in appearance than uPAR^-^ cells. These results stand in stark contrast to the lower respiratory activity of uPAR^+^ cells relative to uPAR^-^ cells.

### Dependence on OXPHOS and mitochondrial substrate-level phosphorylation varied in CSCs and non-stem cancer cells in SCLC

**Fig 4:**
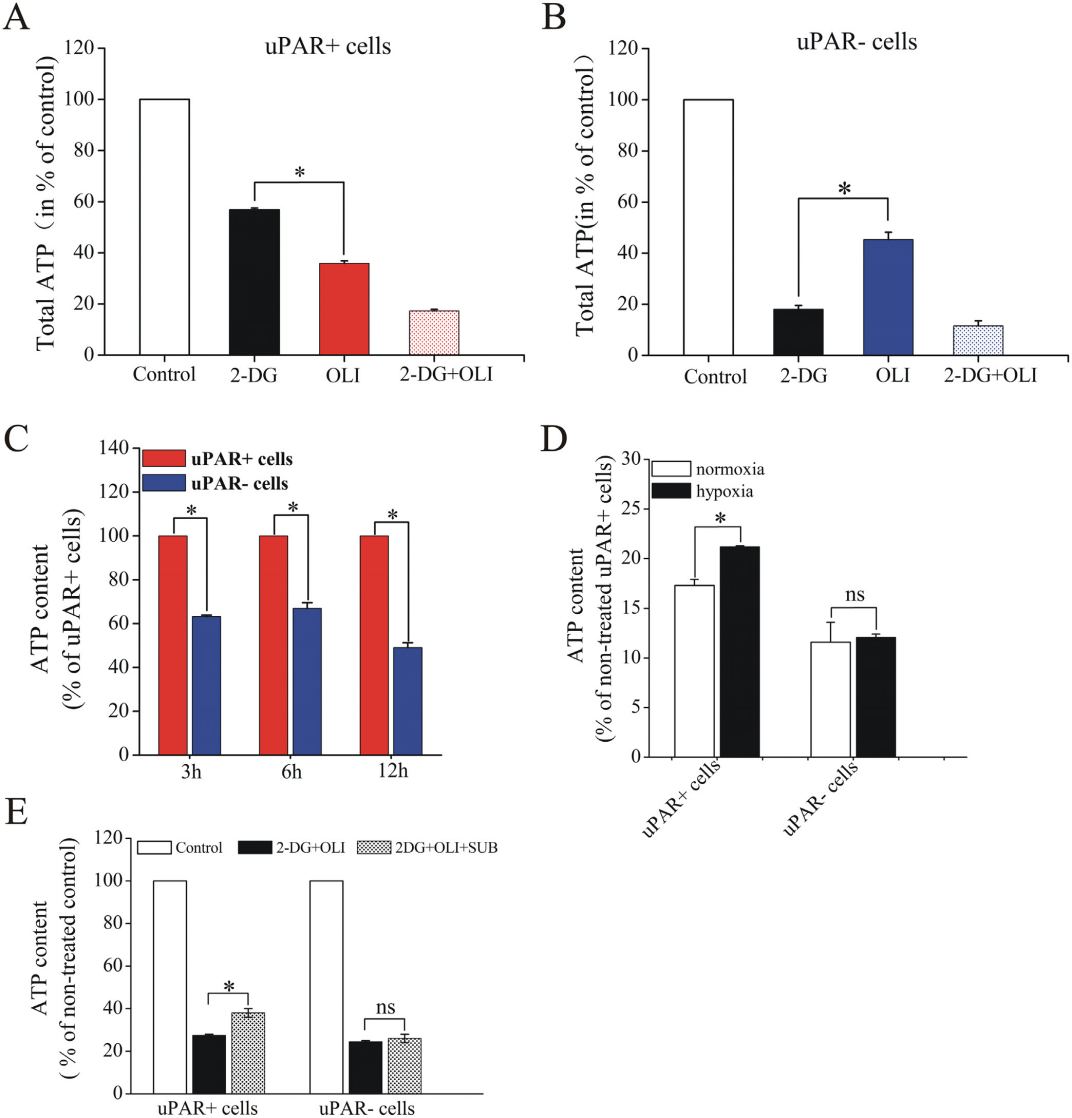
Effect of metabolic inhibitors on ATP levels in CSCs and non-stem cancer cells. (A, B) ATP content of uPAR^+^ cells and uPAR^-^ cells treated with 2-DG, oligomycin (OLI) or a combination of 2-DG and OLI. (C) The effect of combination of 2-DG and OLI for various time on intracellular ATP levels in uPAR^+^ cells and uPAR^-^ cells. (D) The uPAR^+^ cells, not the uPAR^-^ cells could produce more ATP through mitochondrial substrate-level phosphorylation under hypoxic conditions than normoxic conditions (E) The addition of substrates (SUB) could remedy the ATP depletion caused by 2-DG and OLI in uPAR^+^ cells but not in uPAR^-^ cells. Data are expressed as mean±s.e.m. of three independent experiments. Student’s t-test was used to calculate statistical significance. *P<0.05. SUB, substrates (α-ketoglutarate and aspartate). ns, no significance.

To explore the relative importance of OXPHOS and glycolysis in CSCs, we decided to treat both subgroups with different metabolic inhibitors and reanalyze the ATP levels after treatment [15]. 2-DG was a competitive glycolysis inhibitor, and oligomycin was used to inhibit the ATP production via the mitochondrial ATP synthase. As shown in Fig 4A, the ATP levels of uPAR^+^ cells in H446 decreased by 43.2% and 64.1% of the control value when treated with 2-DG and oligomycin, respectively. In uPAR^-^ cells, 2-DG caused an 81.9% ATP drop, whereas the ATP drop induced by oligomycin was 54.7% (Fig 4B). When the effect of oligomycin and 2-DG on ATP levels was compared in two subpopulations, oligomycin had more potent effect on uPAR^+^ cells. By contrast, 2-DG affected uPAR^-^ cells more intensively than uPAR^+^ cells (S3 Fig). The data indicated that the uPAR^+^ cells were more sensitive to OXPHOS inhibition compared with uPAR^-^ cells. To further explore the main energy-generating pathway in CSCs, we calculated the bioenergetic organizations of glycolysis and OXPHOS [28] in uPAR^+^ cells and uPAR^-^ cells. The bioenergetic organizations are heterogeneous and predict differential bioenergetic dependence on glycolysis and OXPHOS for both subpopulations (S4 Fig). These results suggest that CSCs may depend more on OXPHOS rather than glycolysis to meet energy demands. Furthermore, we observed the two cell subpopulations still could produce ATP even though treated with the combination of 2-DG and oligomycin. This implied that these cells may rely on other energy-generating pathways, such as mitochondrial substrate-level phosphorylation during respiratory depression.

Reports have suggested that “forcing” substrate-level phosphorylation to work overtime may be a viable strategy to survive in the energy crisis induced by OXPHOS impairment in yeast [32,33]. To optimize this strategy to SCLC, we inhibited glycolysis and OXPHOS and thus forced cells to produce ATP through mitochondrial substrate-level phosphorylation. To this end, we first set out to determine a low dosage of oligomycin and 2-DG that completely inhibited OXPHOS and glycolysis activity, respectively. Upon treating uPAR^-^ cells with different doses of 2-DG ranging from 5 to 80mM for 3h, the ATP levels of these cells were measured. The ATP was not further decreased by raising 2-DG from 20 to 40 and 80mM, indicating that 20mM 2-DG completely suppressed the ATP production in uPAR^-^ cells (S5A Fig). In the same way, we suggested that 2μg/ml oligomycin could inhibit OXPHOS completely in uPAR^+^ cells (S5B Fig). We observed that uPAR^+^ cells produced more ATP content through substrate-level phosphorylation than do uPAR^-^ cells (Fig 4C). Moreover, the uPAR^+^ cells produced more ATP through substrate-level phosphorylation under hypoxic conditions than under normoxic conditions (Fig 4D). Furthermore, ATP assays showed that α-ketoglutarate/aspartate supplementation to the culture medium could remedy the ATP depletion caused by oligomycin and 2-DG in uPAR^+^ cells. However, this phenomenon was not observed in uPAR^-^ cells (Fig 4E). These observations further indicated that mitochondrial substrate-level phosphorylation in uPAR^+^ cells may be more active than that in the uPAR^-^ cells.

### Oligomycin impaired sphere-forming ability of CSCs

**Fig 5:**
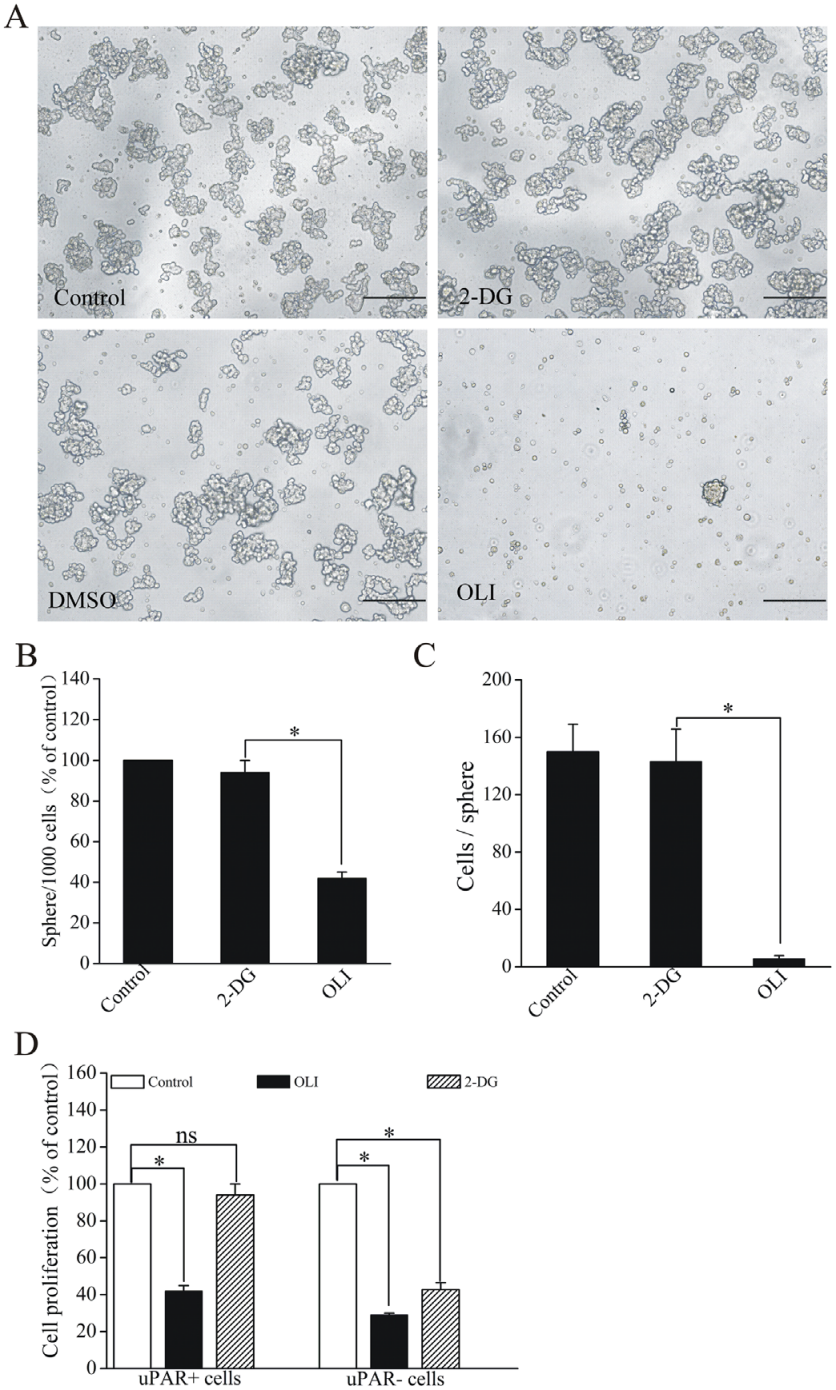
Impairment of oligomycin on sphere-forming and proliferative abilities of CSCs in H446 cells. (A) Representative morphology of spheres maintaining in serum-free medium were established from uPAR^+^ cells treated with oligomycin and 2-DG. (B) Amount of the first-generation spheres generated from uPAR^+^ cells treated with 2-DG and oligomycin. Clonogenic potential of uPAR^+^ cells was reduced by oligomycin treatment. In contrast, 2-DG does not significantly affect the clonogenic potential of CSCs. (C) Number of cells per sphere generated from uPAR^+^ cells treated with 2-DG and oligomycin on the day of 14. (D) Oligomycin inhibits proliferation of both the uPAR^+^ cells and uPAR^-^ cells, however 2-DG affects only uPAR^-^ cells but not uPAR^+^ cells. Data are expressed as mean ± s.e.m. of three independent experiments. Student’s t-test was used to calculate statistical significance. *P<0.05.

Our observations provide evidence that CSCs-enriched uPAR^+^ cells may heavily depend on OXPHOS as their main energy-generating pathway. We then tested whether oligomycin could lead to effective killing of CSCs in SCLC, based on the rationale that oligomycin would deplete the cellular ATP content substantially by inhibiting OXPHOS. Since in vitro clonogenicity is considered as an indicator of the tumor-initiating capability of cancer cells in vivo[5], we tested the effect of oligomycin and 2-DG on the ability of CSCs in H446 to form spheres. 2-DG could not abrogate clonogenicity of CSCs, but oligomycin decreased the number and size of spheres in culture (Fig 5A–5C). Addition of 2-DG to the culture medium showed that inhibition of anaerobic glycolysis severely affected the growth of uPAR^-^ cells but not uPAR^+^ cells. In contrast, oligomycin significantly decreased proliferation of both cell subtypes (Fig 5D).

### Oligomycin suppressed tumor-initiating ability of CSCs in vivo

**Fig 6:**
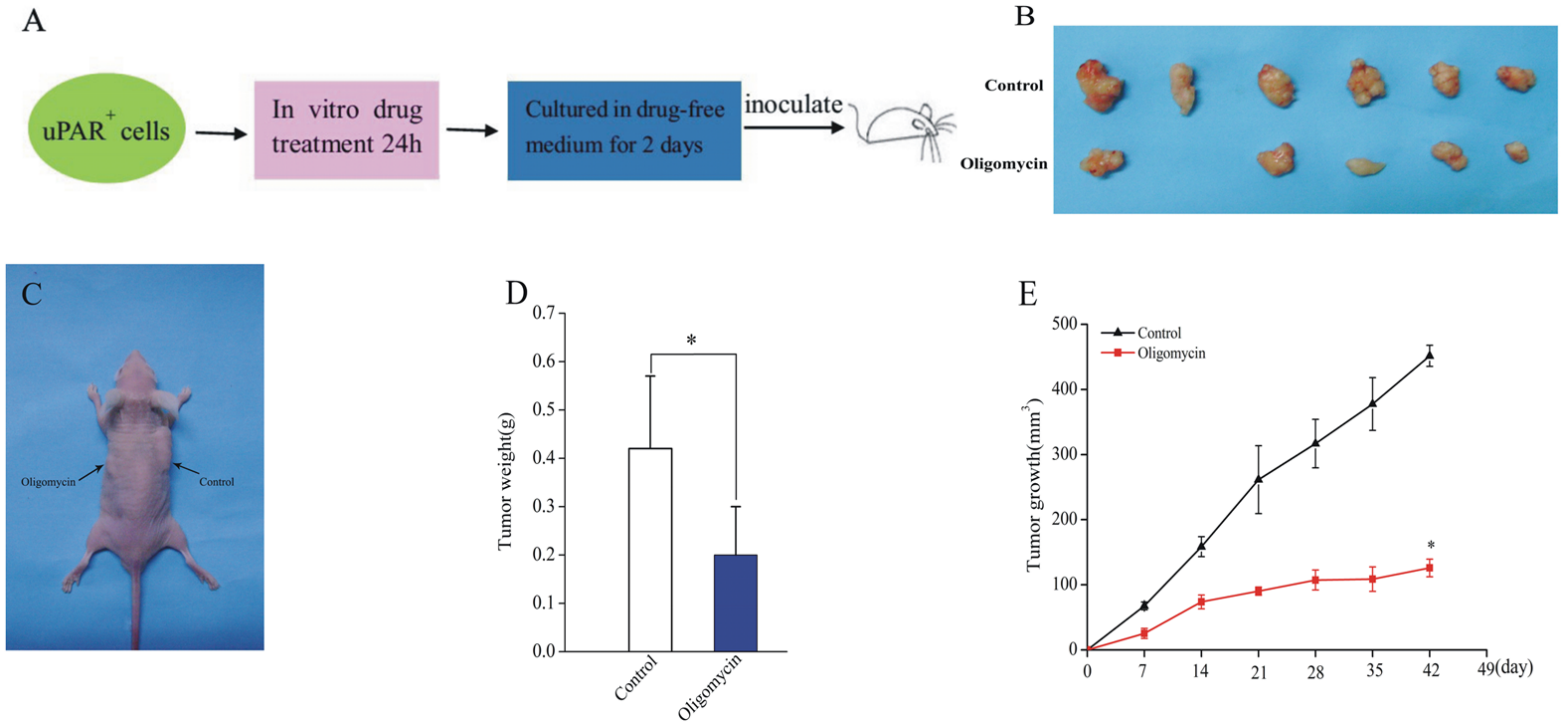
The effect of oligomycin on CSCs tumor formation ability in vivo. (A) Experimental scheme. (B) Representative photograph of tumors formed by the cells treated with oligomycin or control cells. (C) Representative photograph of BALB/cA nude male mice with tumor at 42 days. (D) Tumor weight of xenograft tumors volume of the group treated by oligomycin and control group. (E) Growth curve of xenograft tumors volume of the group treated by oligomycin and control group. Data are expressed as mean ± s.e.m. of six mice. Student’s t-test was used to calculate statistical significance. *P<0.05.

To test the effect of oligomycin to kill the tumor-initiating cells in the CSCs subpopulations, uPAR^+^ cells were treated by the procedure shown in Fig 6A. Then two groups of cells were inoculated subcutaneously BALB/cA nude mice, which were divided into two groups. The mice were then observed for tumor formation and tumor growth without further drug treatment [12]. Six weeks later, group treated by oligomycin and control group formed tumors (Fig 6B and 6C). The group treated by oligomycin had a significantly reduced tumor volume and weight (125.8±13.67 mm^3^, 0.2±0.1g) compared with the control group (451.5±16.23 mm^3^, 0.42±0.15 g) (Fig 6D and 6E). These observations suggest CSCs maintain their stem-like properties by OXPHOS in SCLC cell line H446.

For clinical application, it’s better to take the non-cancer cells into consideration. In our study, the concentration of oligomycin is 2ug/ml, which is far less than the lethal dose[34], and all the mice in experimental group are still in good condition until the end of the experiment. Therefore, we think that the side effect of oligomycin is only a little in our study. In addition, other group has shown that targeted anti-mitochondrial therapy by using oligomycin is effective to arrest MCF-7 spheroid growth without apparent effect on normal epithelial breast tissue at similar doses[35]. But in view of time and our original intention, we tend to illustrate the distinct metabolic phenotypes of the bulk of the cancer cells and CSCs. Therefore, we regard CSCs and non-stem cancer as research objects in this paper.

## Discussion

Until now, the vast majority of studies in this field have focused on the bulk tumor cells, which show increased glycolysis even in the presence of oxygen in a variety of tumors [17]. However, there is increasing evidence indicating that the metabolic state of CSCs differs from non-stem cancer cells [36–38]. Little is known about the metabolic properties of CSCs in SCLC so far. Here, we showed that CSCs resided in a state showing low metabolic activity in SCLC. Our results implied that CSCs-enriched uPAR^**+**^ cells were dormant subpopulations in SCLC. The cellular dormancy could bring advantages for tumor cells. On the one hand, most of current therapies is targeting the proliferating tumor cells. Thus the dormant CSCs may survive targeted therapies and be responsible for tumor recurrence. On the other hand, this relatively dormant state likely makes CSCs persist, even under highly stressful conditions such as low nutrient or oxygen levels[16]. The current research provides chances to understand the complexities of tumor dormancy, but the mechanism of this process needs further investigation.

miRNAs play essential regulatory roles in various biological processes, such as metabolism [27]. Previous work from our laboratory compared the miRNA expression profiles of CSCs and non-stem cancer cells from H446 using miRNA array including 1212 mature miRNAs. In the array, it was found that 86 miRNAs were differentially expressed between CSCs and non-stem cancer cells (P<0.01), including 48 upregulated miRNAs and 38 downregulated miRNAs in CSCs. Among 86 differentially expressed miRNAs, 18 of the 48 upregulated miRNAs and 20 of the 38 downregulated miRNAs showed at least a 4-fold changes in CSCs relative to non-stem cancer cells[29]. Therefore, our next work is to explore the effect of miRNAs on metabolic state of CSCs in SCLC.

In this study, we showed that CSCs showed preferential use of OXPHOS over glycolysis to meet their energy demand. To the best of our knowledge, CSCs reside in hypoxic environments [39]. Why are CSCs still highly reliant on OXPHOS in hypoxic microenvironment? We think the reasons are as follows. Firstly, oxygen concentration in a hypoxic microenvironment has been suggested to be within the scope of 8–57μM [4,40–42], which is higher than the limiting concentration for the O_2_ dissociation constant for cytochrome c oxidase [43]. Therefore, CSCs still could heavily rely on OXPHOS even under hypoxic conditions. Secondly, there is a metabolic symbiosis between two subpopulations of cancer cells with distinct dependencies in energy-producing pathways [44,45]. Glycolytic cancer’s byproduct lactate can be used as an important metabolite for OXPHOS by cancer subsets which are dependent on mitochondrial metabolism. The metabolic symbiosis enables cancer cells to make full use of available resources[45]. Thirdly, Otto Warburg’ work has shown that highly proliferative cells often preferentially perform glycolysis over OXPHOS [17]. Through glycolysis, some glucose must be diverted to the acetyl-CoA and NADPH needed for macromolecular synthesis [46]. However, this is not necessary for CSCs which sustain in a state showing low metabolic activity.

We also found that CSCs could generate ATP through the mitochondrial substrate-level phosphorylation, which is the only way for production of high-energy phosphates in the absence of oxygen or when the electron transport chain is blocked[47]. Succinyl-CoA synthetase (SCS) is the only mitochondrial enzyme which catalyze the mitochondrial substrate-level phosphorylation [48]. The succinyl-CoA provided by α-ketoglutarate dehydrogenase complex (KGDHC) can serve as the substrate of the mitochondrial substrate-level phosphorylation when the electron transport chain is dysfunctional [49]. Previous work has shown that Inorganic phosphate (Pi) can directly activate dehydrogenases, including KGDHC [49] and SCS [48]in citric acid cycle. There is still sufficient Pi, KGDHC and SCS in CSCs, though the electron transport chain is damaged. Therefore, CSCs treated with 2-DG and oligomycin still maintain themselves on substrate-level phosphorylation in citric acid cycle. In addition, we showed that α-ketoglutarate/aspartate supplementation to the culture medium remedy the ATP depletion caused by oligomycin and 2-DG in CSCs. Because α-ketoglutarate is transferred to the mitochondrial matrix [50], where it is converted first to succinyl-CoA, then produced ATP through mitochondrial substrate-level phosphorylation. Its byproduct NADH is used to transform oxaloacetate (provided as its precursor aspartate) into malate, which in turn, may leave the mitochondrial matrix through the oxoglutarate carrier that exchanges it for α- ketoglutarate[32]. Currently, we only concentrated on the change of ATP levels in the study, next we intended to investigate the relative expression levels of genes, mRNAs and protein of SCS to further compare the mitochondrial substrate-level phosphorylation in CSCs and non-stem cancer cells.

Taken together, we have indicated that CSCs mainly rely on OXPHOS for energy generation. Therapeutic strategies based on the assumption that tumors are preferentially reliant on glycolysis may need to be reconsidered [16]. We hope that the further understanding of the metabolic state of CSCs in SCLC would yield improved therapeutic strategies.

## Conclusions

CSCs are metabolically inactive tumor subpopulations which sustain in a state showing low metabolic activity. CSCs display higher dependency on oxidative phosphorylation and mitochondrial substrate-level phosphorylation compared with non-stem cancer cells. Oxidative phosphorylation inhibition may result in effective killing of CSCs in small cell lung cancer cell line H446.

## Supporting Information

S1 Fig Isolation of uPAR^+^ cells and uPAR^-^ cells from a small cell lung cancer cell line H446. (TIF)

S2 Fig Effect of glycolysis inhibitor on lactate production in uPAR^+^ cells and uPAR^-^ cells. (TIF)

S3 Fig Comparison of ATP content of uPAR^+^ cells and uPAR^-^ cells treated by the same metabolic inhibitor(s). (TIF)

S4 Fig Determination of bioenergetic organizations of uPAR^+^ cells and uPAR^-^ cells. (TIF)

S5 Fig Determination of a low dosage of oligomycin and 2-DG that completely inhibited OXPHOS and glycolysis activity. (A) ATP levels in uPAR^-^ cells treated with various concentrations of 2-DG. (B) ATP levels in uPAR^+^ cells treated with various concentrations of OLI (oligomycin). ns, no significance.
(TIF)
