# Supplementary figures and images for: Cancer Stem Cells in Small Cell Lung Cancer Cell Line H446: Higher Dependency on Oxidative Phosphorylation and Mitochondrial Substrate-Level Phosphorylation than Non-Stem Cancer Cells
Source: PLoS One. 2016 May 11;11(5):e0154576. doi: 10.1371/journal.pone.0154576 (PMC4863974; doi:10.1371/journal.pone.0154576)

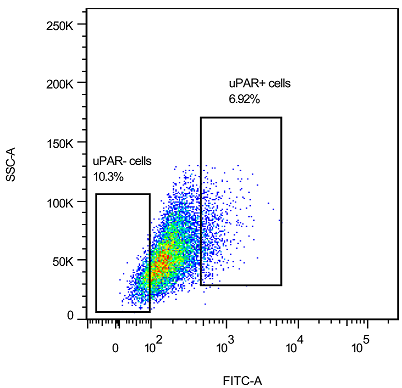

Supplement: S1 Fig — (TIF) [file pone.0154576.s001.tif]

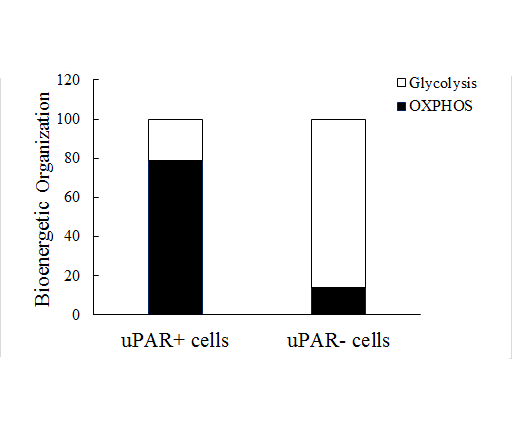

Supplement: S4 Fig — (TIF) [file pone.0154576.s004.tif]
